# The Royal College of Surgeons of England

**Published:** 1898-04-23

**Authors:** 


### THE ROYAL COLLEGE OF SURGEONS OF ENGLAND.

At the quarterly meeting of the Council the Jacksonian prize for 1897 was awarded to Mr. Percy Furnival, of St. Bartholomew's Hospital. At the recommendation of the Committee of Management, certain institutions were added to the list of those recognised as places of instruction in chemistry, physics, and chemistry. It was also resolved that the regulations be altered so that the first of the five years of the curriculum may be spent in obtaining instruction in chemistry, physics, practical chemistry, and biology, either at a recognised institution or a recognised medical school, and that candidates be required to spend four winter and four summer sessions, from the date of passing in those subjects at a recognised school and hospital before entering for examination in medicine and surgery. We thus see the hospital curriculum again cut down to four years—or in practice to two years actually in hospital—the whole of the lengthened curriculum which was intended to increase the student's practical knowledge of disease, being thus entirely appropriated by preliminary studies. A by no means satisfactory conclusion. The Council then took into consideration the petition presented by certain Fellows and members of the college, claiming that the right of electing the representative of the College to the General Medical Council is, by Section 7 of the Medical Act, 1886, vested in the Fellows and members of the College, and calling upon the Council to make arrangements to hold an election, and enable the Fellows and members to choose a representative. It was resolved that the petitioners be informed that the Council see no reason to alter the mode of election, which has been in force for the last forty years, and consider that no useful purpose would be served by receiving a deputation from the petitioners in reference to their claims. We can hardly imagine that the petitioners will be stopped in their career by such a resolution. The course adopted by the Council was, however, the only one which was open to them. It was not to be expected that they would stultify themselves by admitting that for forty years they had been acting wrongly under an erroneous interpretation of the Act. The interpretation of an Act of Parliament is clearly a matter for a court of law, and the sooner this question is decided by an amicable action the better.

